# Clinical Utility of Intracellular Organisms in Gram-Stained Sputum Among Patients With Methicillin-Resistant Staphylococcus aureus Pneumonia

**DOI:** 10.7759/cureus.112615

**Published:** 2026-07-13

**Authors:** Yoriko Herai, Toshibumi Taniguchi, Sho Yokota, Hiroshi Yoshikawa, Misuzu Yahaba, Shota Murata, Hidetoshi Igari

**Affiliations:** 1 Department of Infectious Diseases, Chiba University Hospital, Chiba, JPN; 2 Department of Allergy and Immunology, Graduate School of Medicine, Chiba University, Chiba, JPN; 3 Division of Laboratory Medicine, Chiba University Hospital, Chiba, JPN

**Keywords:** gram-stained sputum, intracellular organisms, methicillin-resistant staphylococcus aureus, phagocytosis, pneumonia

## Abstract

Introduction: Sputum cultures may detect bacteria that are not the true causative pathogens of pneumonia. Gram-stained sputum has demonstrated utility in the diagnosis and treatment of pneumonia.

Gap statement: However, the clinical significance of intracellular organisms (ICOs) observed in Gram-stained sputum has not been fully elucidated.

Aim: This study aimed to evaluate whether the presence of ICOs morphologically consistent with *Staphylococcus* species in Gram-stained sputum specimens is associated with the diagnosis of methicillin-resistant *Staphylococcus aureus* (MRSA) pneumonia and the clinical response to anti-MRSA therapy.

Methodology: A retrospective matched case-control study was conducted between January 2010 and December 2022. Patients who underwent sputum smear and culture testing at Chiba University Hospital were included.

Results: Of the 1,075 patients who underwent sputum testing, 65 patients with ICO were identified and matched 1:1 with 65 patients without ICO for analysis. Among these, 30 patients (46.2%) in the ICO-positive group were diagnosed with MRSA pneumonia, compared with 21 patients (32.3%) in the ICO-negative group. The risk difference was 13.9% (bootstrapped 95% CI -3.8% to 30.5%), and the difference was not statistically significant (p = 0.15). Furthermore, among patients with pneumonia, the presence of ICO was not associated with a statistically significant difference in the clinical outcomes of anti-MRSA therapy (p = 0.32; bootstrapped 95% CI, -0.434 to 0.292).

Conclusion: The presence of ICOs in sputum samples was not significantly associated with MRSA pneumonia and may have limited utility in predicting the clinical response to anti-MRSA therapy.

## Introduction

When bacteria breach the body’s physical barriers, such as mucosal surfaces and the host microbiota, phagocytic cells are activated. Innate immunity is then initiated within hours of bacterial invasion, typically more rapidly than adaptive immunity [1].

Generally, Gram stains may reveal the presence of microorganisms within white blood cells (WBCs), including leukocytes. These microorganisms are referred to as intracellular organisms (ICO) and may result from either phagocytosis or microbial growth. In cases of ventilator-associated pneumonia (VAP), an ICO percentage exceeding 1.5%-2% in bronchoalveolar lavage fluid (BALF), suggesting a high likelihood of bacterial pneumonia, has been reported [2,3]. Gram-stained sputum has been shown to be useful in the diagnosis and treatment of pneumonia; however, the clinical significance of ICO identified in Gram-stained sputum remains poorly investigated.

Notably, bacteria identified through sputum culture may not necessarily represent the true causative pathogens of pneumonia. This study focused on methicillin-resistant Staphylococcus aureus (MRSA) and aimed to investigate whether the presence of ICO aids in the diagnosis of MRSA pneumonia and predicts the clinical response to anti-MRSA therapy.

This article was previously posted to the Access Microbiology preprint server on May 28, 2026.

## Materials and methods

Study design and participants

This retrospective matched case-control study was conducted at Chiba University Hospital, a tertiary-care academic medical center in Japan. The study period extended from January 2010 to December 2022. We reviewed all patients who underwent both sputum Gram staining and sputum culture because of respiratory symptoms or suspected pneumonia. Among these patients, those with MRSA isolated from sputum cultures were eligible for inclusion.

**Figure 1:**
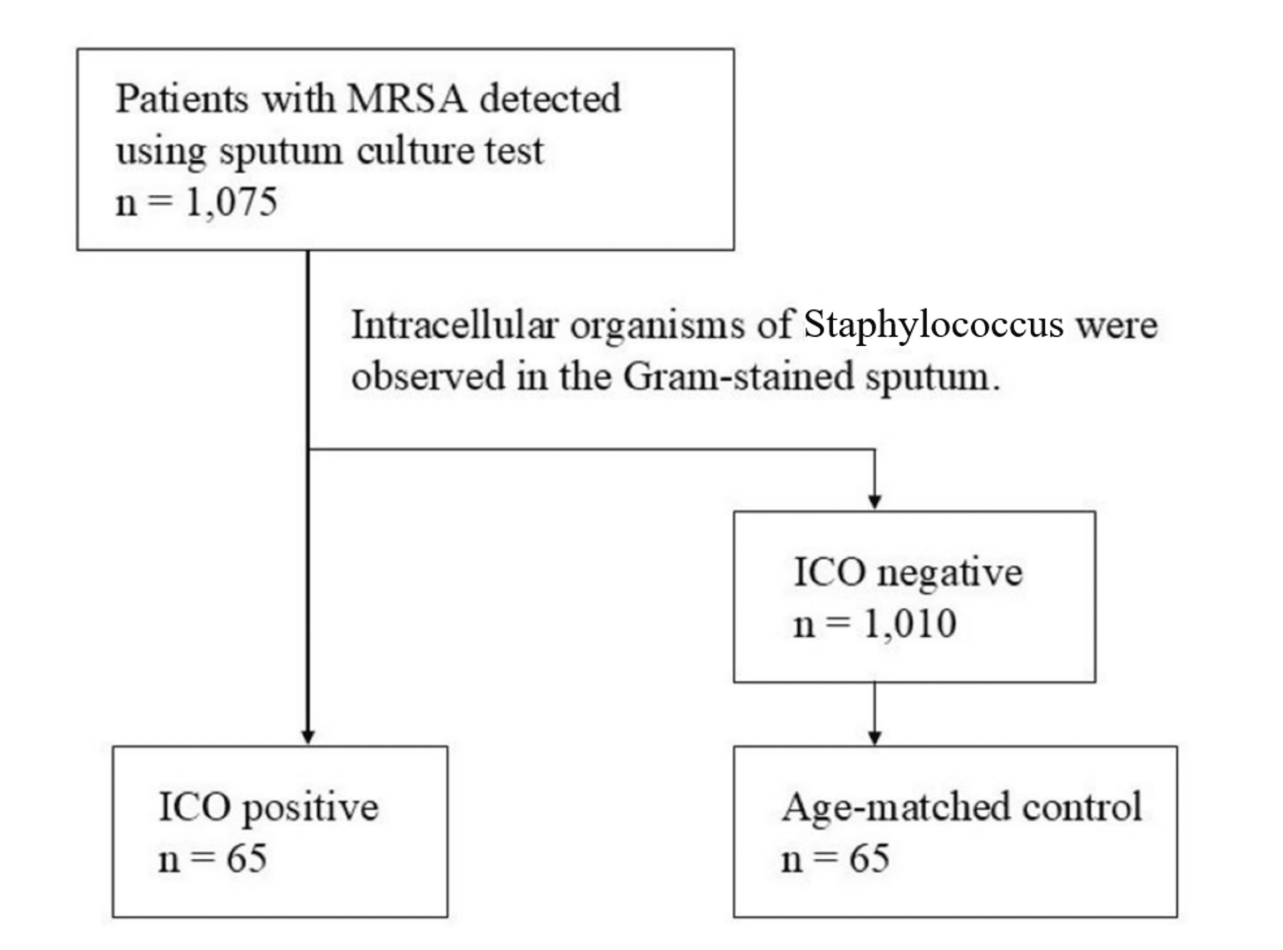
Flowchart of patients in whom MRSA was detected in sputum culture tests. MRSA: methicillin-resistant *Staphylococcus aureus,* ICOs: intracellular organisms.

Clinical and laboratory data were retrospectively extracted from the electronic medical record system. Patients in whom ICOs morphologically consistent *with Staphylococcus* species were identified on Gram-stained sputum specimens were classified as the ICO-positive group. For each ICO-positive patient, one ICO-negative patient was selected using 1:1 age- and sex-matching. Only patients with complete demographic, clinical, microbiological, and laboratory data were included in the analysis. The study flow is shown in Figure 1.

Definition of ICO

**Figure 2:**
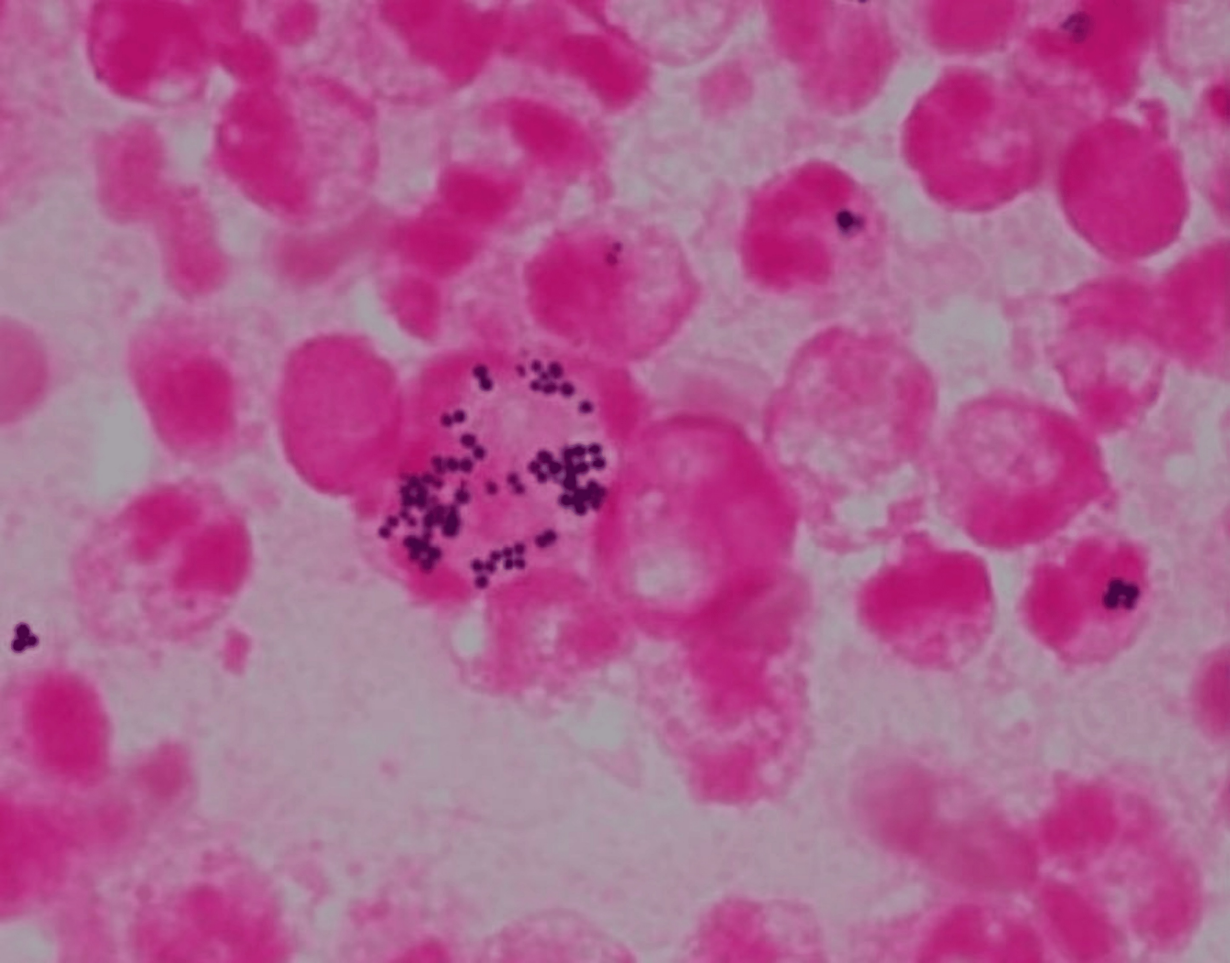
Representative image of ICO on Gram-stained sputum. ICO: intracellular organism.

In this study, ICO was defined as the presence of *Staphylococcus* organisms within the cytoplasm of polymorphonuclear leukocytes observed on Gram-stained sputum specimens [4]. Gram-stained slides were routinely evaluated by experienced microbiologists in the Division of Laboratory Medicine at Chiba University Hospital according to standard laboratory procedures. Sputum quality was assessed using the Q score system [5]. The presence of ICO was determined morphologically by identifying Gram-positive cocci within the cytoplasm of leukocytes (Figure 2). No predefined threshold for the number of microscopic fields examined or the number of intracellular bacteria required for a positive result was applied.

To minimize observational bias, the microbiologists who interpreted the Gram-stained specimens were unaware of the specific objectives of the present study because the evaluations were performed as part of routine clinical practice. Predominance of MRSA on Gram-stained sputum smears was defined as a proportion of bacteria observed microscopically that exceeded that of other bacterial species identified simultaneously in culture. After the organism was morphologically identified as *S. aureus*, it was diagnosed as MRSA based on culture and antimicrobial susceptibility testing.

Demographic, clinical, and laboratory data collection

Data were extracted from electronic medical records, including demographic information, underlying medical conditions, laboratory results, and medication histories within the 90 days preceding sputum culture collection.

Definition of pneumonia

The diagnosis of pneumonia was established when all of the following criteria were met [6]. First, patients were required to have at least one clinical sign or symptom suggestive of a lower respiratory tract infection, including cough, purulent sputum production, abnormal auscultatory findings such as crackles or diminished breath sounds, worsening dyspnea or tachypnea, or an axillary temperature of at least 37°C. Second, newly developed pulmonary infiltrates compatible with pneumonia had to be identified on chest radiography or computed tomography. Third, patients were required to demonstrate at least one objective indicator of systemic inflammation or respiratory compromise, including a WBC count ≥10,000/mm³, an elevated CRP level, or hypoxemia, defined by an arterial oxygen tension <60 Torr or peripheral oxygen saturation <90%.

Pneumonia cases were further classified according to the site of acquisition. Community-acquired pneumonia was defined as pneumonia developing outside healthcare settings. Hospital-acquired pneumonia (HAP) was defined as pneumonia occurring at least 48 hours after hospital admission and included cases of ventilator-associated pneumonia (VAP). VAP was defined as pneumonia developing at least 48 hours after endotracheal intubation and the initiation of mechanical ventilation.

Evaluation of antimicrobial efficacy

Clinical efficacy of antimicrobial therapy was evaluated based on changes in clinical symptoms, laboratory parameters, and radiographic findings after treatment initiation. Treatment was considered clinically effective when at least three of the following five criteria were met: improvement or complete resolution of clinical symptoms; reduction of body temperature to below 37°C; improvement in chest radiographic findings to ≤70% of the baseline severity score; reduction of the WBC count to <9,000/mm³; and reduction of the CRP level to <30% of the pretreatment value. These criteria were adapted from previously published studies evaluating treatment responses in bacterial pneumonia [7].

Statistical analysis

Continuous variables with normal distributions were presented as means with standard deviations, as determined by the Kolmogorov-Smirnov test. Data that did not follow a normal distribution were summarized as medians with interquartile ranges. Categorical variables were expressed as frequencies and proportions. To compare continuous variables between case patients and control subjects, the paired t-test was used for normally distributed data, whereas the Mann-Whitney U test was used for non-normally distributed data. For comparisons of categorical variables between the two groups, Fisher’s exact test was used when the expected cell count was <20, whereas Pearson’s chi-square test was used when the expected cell count was ≥20. Statistical significance was set at a p-value < 0.05. Differences between groups and their 95% CIs were estimated using nonparametric bootstrapping with 2,000 resamples. R version 4.1.2 was used for all statistical analyses (R Foundation for Statistical Computing, Vienna, Austria, https://www.R-project.org/) [8].

## Results

Clinical characteristics of the enrolled patients

In this study, a total of 1,075 patients were included, of whom 65 were classified as ICO-positive. Within the ICO-positive group, a higher proportion of patients had a documented history of antimicrobial therapy within the 90 days preceding Gram staining (Table 1).

**Table 1:** Characteristics of ICO-positive results compared with ICO-negative results. *Sputum characteristics were evaluated using the Q-score, with higher numbers indicating better quality. **Predominance is defined as the highest proportion of bacteria detected. ICO: intracellular organisms, DM: diabetes mellitus, CAP: community-acquired pneumonia, HAP: hospital-acquired pneumonia, VAP: ventilator-associated pneumonia, WBC: white blood cell, CRP: C-reactive protein, IQR: interquartile range.

	ICO-positive, n = 65	ICO-negative, n = 65	p-value	Available data for ICO-positive patients, n	Available data for ICO-negative patients, n
Age, y (IQR)	64 (50-74)	67 (57-74)	0.48	65	65
Male, n (%)	44 (67.7)	44 (67.7)	1	65	65
Underlying diseases					
DM, n (%)	7 (10.8)	12 (18.5)	0.21	65	65
Malignant solid tumor, n (%)	32 (49.2)	31 (47.7)	0.73	65	65
Use of glucocorticoids, n (%)	7 (10.8)	10 (15.4)	0.44	65	65
Use of immunosuppressive agents, n (%)	1 (1.5)	1 (1.5)	1	65	65
Type of pneumonia					
CAP, n (%)	4 (6.2)	6 (9.2)	0.74	65	65
HAP excluding VAP, n (%)	18 (27.7)	14 (21.5)	0.54	65	65
VAP, n (%)	8 (12.3)	2 (3.1)	0.16	65	65
Laboratory findings					
WBC,/µL (IQR)	9,601 (6,275-10,225)	9,211 (6,000-10,200)	1	64	61
CRP, mg/dL (IQR)	7.81 (3.08-10.37)	8.25 (1.63-10.70)	0.99	64	61
Sputum					
Q score*	7 (10.8)	28 (43.1)	<0.05	65	65
0					
1	9 (13.8)	8 (12.3)		65	65
2	26 (55.4)	9 (13.8)		65	65
3	23 (35.4)	20 (30.8)		65	65
Predominance of detected bacteria**, n (%)	60 (92.3)	26 (40.0)	<0.05	65	65
Use of antibiotics within 90 days, n (%)	23 (35.4)	37 (56.9)	<0.05	65	65
Alive and discharged from hospital at 30 days, n (%)	15 (23.1)	31 (47.7)	<0.05	64	65
90-day mortality, n (%)	41 (71.9)	50 (86.2)	0.06	57	58

Additionally, a greater proportion of patients in the ICO-positive group were discharged after 30 days. However, no statistically significant difference in 90-day survival was observed compared with the ICO-negative group. Regarding sputum characteristics, the ICO-positive group had higher Q-scores and a higher prevalence of MRSA predominance in sputum cultures. Apart from these findings, no significant differences were observed in patient demographics or baseline characteristics between the ICO-positive and ICO-negative groups.

Comparison of infection and no infection groups with emphasis on ICO

In the ICO-positive group, 30 of 65 patients (46.2%) were diagnosed with pneumonia, whereas 21 of 65 patients (32.3%) in the ICO-negative group were diagnosed with pneumonia (Table 2). The risk difference was 13.9% (bootstrapped 95% CI, -3.8% to 30.5%), and the difference was not statistically significant (p = 0.15).

**Table 2:** Frequency of ICO and pneumonia. No statistically significant difference was found for the frequency of pneumonia between the ICO-positive and ICO-negative patient groups (p = 0.15). ICO: intracellular organism.

	Pneumonia		Total	p-value	95% bootstrap CI	
	+	-			Lower	Upper
ICO-positive	30	35	65	0.15	-0.038	0.305
ICO-negative	21	44	65			

Efficacy of anti-MRSA drugs in pneumonia

We evaluated whether the presence of ICO was associated with the clinical efficacy of anti-MRSA therapy in patients with pneumonia. There was no statistically significant difference in treatment efficacy between patients with and without ICO (p = 0.32; bootstrapped 95% CI, -43.4% to 29.2%) (Table 3).

**Table 3:** Efficacy of anti-MRSA drugs against pneumonia evaluated separately as ICO-positive and ICO-negative. The results suggest no statistically significant difference in the efficacy of anti-MRSA drugs, whether ICO was present or not. ICO: intracellular organism, MRSA: methicillin-resistant *Staphylococcus aureus.*

Pneumonia	Administration of anti-MRSA drugs	Effective	p-value	95% bootstrap confidence interval	
				Lower
ICO-positive (n = 30)	24 (80.0%)	12 (50.0%)	0.72	-0.434
ICO-negative (n = 21)	10 (47.6%)	6 (60.0%)		

Anti-MRSA agents were selected based on the results of antimicrobial susceptibility testing and included vancomycin, teicoplanin, trimethoprim-sulfamethoxazole, and linezolid.

## Discussion

When bacteria invade the human body, neutrophils and macrophages, key components of the innate immune system, play a pivotal role by engulfing and phagocytosing invading pathogens [9]. Innate immune activity is presumed when ICOs are detected in Gram-stained specimens from infected sites.

Gram stains of sputum samples are affordable, noninvasive, and readily accessible diagnostic tools that can immediately identify the causative bacteria, particularly when performed by experienced observers on high-quality specimens. Several studies have reported that microorganisms observed in Gram-stained specimens are valuable for identifying the causative agents of pneumonia [10,11]. However, our comprehensive literature review found that the utility of ICO observed on Gram stains has primarily been reported in the context of BALF for the diagnosis of VAP [2,3]. In our study, the presence of ICO was not significantly associated with the diagnosis of pneumonia (Table 2).

Previous reports have indicated that the frequency of ICO differs depending on the bacterial species involved [4]. For *S. aureus*, the sensitivity and specificity of sputum Gram stains were reported to be 9.1% and 100%, respectively [12]. We initially hypothesized that the presence of ICO might be influenced by sputum specimen quality, potentially favoring the diagnosis of pneumonia in the ICO-positive group. However, our findings revealed no statistically significant difference. In particular, the low sensitivity of sputum Gram staining for identifying MRSA pneumonia may explain why ICO did not demonstrate clear diagnostic utility in this study.

Analysis using the clone library method identified 13 cases of pneumonia in which *S. aureus* (subsequently confirmed as MRSA by culture) was detected, and anti-MRSA agents were subsequently administered. However, seven of these cases were refractory to anti-MRSA therapy [7]. In our study, the presence of ICO was not significantly associated with the clinical efficacy of anti-MRSA therapy (Table 3).

This study had several limitations. First, as this was a retrospective study, certain essential clinical data, such as 90-day mortality, were not consistently available for all patients. Second, the absence of a standardized definition of ICO may have resulted in interobserver variability in ICO assessment. Third, because there is no established gold standard for diagnosing MRSA pneumonia, the diagnosis was based on a combination of clinical symptoms, laboratory findings, and radiologic findings. Fourth, unlike BALF, sputum Gram staining is not a quantitative test, making quantitative assessment difficult. Fifth, the study may have been limited by its sample size, as the optimal number of cases was not predetermined. In addition, because this was a retrospective single-center study, selection bias cannot be completely excluded. Although cases and controls were matched by age and sex, residual confounding due to unmeasured clinical factors may have influenced the observed associations. Finally, our findings may not be generalizable to institutions with different patient populations, microbiological epidemiology, or laboratory practices. Therefore, multicenter prospective studies are warranted to validate our findings.

## Conclusions

To the best of our knowledge, few studies have evaluated the clinical significance of ICO in MRSA-positive sputum samples with respect to the diagnosis of pneumonia and response to antimicrobial therapy. In summary, the presence of ICO in sputum samples was not significantly associated with MRSA pneumonia and may have limited utility in guiding antimicrobial therapy.
